# Detection of genomic regions underlying milk production traits in Valle del Belice dairy sheep using regional heritability mapping

**DOI:** 10.1111/jbg.12552

**Published:** 2021-05-20

**Authors:** Anna Maria Sutera, Marco Tolone, Salvatore Mastrangelo, Rosalia Di Gerlando, Maria Teresa Sardina, Baldassare Portolano, Ricardo Pong‐Wong, Valentina Riggio

**Affiliations:** ^1^ Dipartimento Scienze Veterinarie Università di Messina Messina Italy; ^2^ Dipartimento Scienze Agrarie, Alimentari e Forestali Università degli Studi di Palermo Palermo Italy; ^3^ The Roslin Institute and R(D)SVS Easter Bush Campus Midlothian UK; ^4^ Centre for Tropical Livestock Genetics and Health (CTLGH) The Roslin Institute University of Edinburgh Easter Bush Campus Edinburgh UK

**Keywords:** dairy sheep, milk production traits, region heritability mapping, SNP, Valle del Belice breed

## Abstract

The aim of this study was to identify genomic regions underlying milk production traits in the Valle del Belice dairy sheep using regional heritability mapping (RHM). Repeated measurements for milk yield (MY), fat percentage and yield (F% and FY) and protein percentage and yield (P% and PY), collected over a period of 6 years (2006–2012) on 481 Valle del Belice ewes, were used for the analysis. Animals were genotyped with the Illumina 50k SNP chip. Variance components, heritabilities and repeatabilities within and across lactations were estimated, fitting parity, litter size, season of lambing and fortnights in milk, as fixed; and additive genetic, permanent environment within and across lactations, flock by test‐day interaction and residual as random effects. For the RHM analysis, the model included the same fixed and random effects as before, plus an additional regional genomic additive effect (specific for the region being tested) as random. While the whole genomic additive effect was estimated using the genomic relationship matrix (GRM) constructed from all SNPs, the regional genomic additive effect was estimated from a GRM matrix constructed from the SNPs within each region. Heritability estimates ranged between 0.06 and 0.15, with repeatabilities being between 0.14 and 0.24 across lactations and between 0.23 and 0.39 within lactation for all milk production traits. A substantial effect of flock‐test‐day on milk production traits was also estimated. Significant genomic regions at either genome‐wide (*p* < .05) or suggestive (i.e., one false positive per genome scan) level were identified on chromosome (OAR) 2, 3 and 20 for F% and on OAR3 for P%, with the regions on OAR3 in common between the two traits. Our results confirmed the role of *LALBA* and *AQP* genes, on OAR3, as candidate genes for milk production traits in sheep.

## 1 INTRODUCTION

The Mediterranean Basin countries host approximately 46% of the total world sheep milk production (Carta et al., 2009). The dairy sheep industry is usually based on local breeds, which are very well adapted to their production systems and environments and can indeed guarantee income, employment and economic viability in areas where production alternatives are scarce or non‐existent. Milk production is the main trait affecting profitability of dairy sheep industry (Riggio, 2012). Traditionally, the genetic control of complex traits (such as milk production) in livestock has been studied without identifying the genes or gene variants underlying observed variation, with selection on the basis of estimated breeding values calculated from phenotypic and pedigree information (Goddard & Hayes, 2009). However, standard breeding programs are not very efficient in sheep breeds, due to several limitations in sheep farming systems. Technical‐ and infrastructural‐related issues are indeed the greatest bottlenecks in genetic improvement programs for sheep breeds. Small flock sizes, poor pedigree and performance recording, lack of clear breeding goals, lack of or poor infrastructures are all factors that contribute to the low participation of farmers in breeding schemes, which in turn makes achieving within‐breed genetic improvement highly challenging (Riggio, 2012). Moreover, genetic gain is hampered by the relatively high costs of large‐scale phenotyping and the logistic constraints of artificial insemination (Carta et al., 2009). Therefore, it would be important to identify genes affecting milk production traits to better understand their genetics and speed up genetic improvement in future generations, overcoming some of these limitations.

Several quantitative trait loci (QTL) for milk production traits in sheep have been identified using both microsatellite markers (Arranz & Gutiérrez‐Gil, 2012; Barillet et al., 2005; Diez‐Tascón et al., 2001; Gutiérrez‐Gil et al., 2009) and single nucleotide polymorphism (SNP) arrays (García‐Gámez, Gutiérrez‐Gil, Sahana, et al., 2012; Li et al., 2020; Sutera et al., 2019) as well as copy number variations (Di Gerlando et al., 2019). However, little consensus has emerged from these studies, probably due to the fact that they are very diverse in terms of methodologies, statistical approaches and sheep breeds.

Moreover, GWAS studies have generally failed to explain most of the known genetic variation influencing complex traits (Kemper et al., 2011; Manolio et al., 2009). One of the shortcomings of a standard GWAS study is the large number of statistical tests performed, which usually requires very stringent thresholds to be applied to avoid spurious associations. Such rigorous thresholds minimize false‐positive associations but also lead to many false negatives since SNPs with small effects or incomplete linkage disequilibrium (LD) with the causative mutation will fail to pass the stringent statistical threshold and remain undetected (Hayes, 2013). Attempts to increase the power of GWAS have focused on increasing the number of observations for each experiment and the density of SNP arrays. Without increasing the number of observations, power could be gained by testing a cumulative effect of multiple variants in a given region of a genome rather than testing each variant individually (Al Kalaldeh et al., 2019), increasing the likelihood of capturing the complete effect of a QTL (Lee et al., 2014). An alternative method, called regional heritability mapping (RHM), has been proposed as a better approach to capture more of the underlying genetic effects (Nagamine et al., 2012). RHM uses a genomic relationship matrix (GRM) between individuals based on all SNPs found in short segments/regions of the genome to estimate the variance of the trait explained by such regions, allowing to potentially detect regions containing multiple variants that individually contribute too little variance to be detected by standard GWAS. The use of RHM, assessed by simulation on full sequence data, detected a larger number of QTL than GWAS did, although QTLs individually explained a slightly smaller amount of genetic variance (Caballero et al., 2015). Even though the RHM method has received increasing attention in humans (Shirali et al., 2016) as well as livestock (Al Kalaldeh et al., 2019; Matika et al., 2016; Raphaka et al., 2017; Riggio et al., 2013; Sánchez‐Molano et al., 2019), to the best of our knowledge, it has not yet been applied to the study of milk production traits in dairy sheep. The objective of this study was, therefore, to identify loci and/or genomic regions affecting milk production traits (i.e., milk yield (MY), fat yield (FY), fat percentage (F%), protein yield (PY) and protein percentage (P%)) in Valle del Belice dairy sheep, using the RHM approach.

## 2 MATERIAL AND METHODS

### 2.1 Ethics statement

In this study, the procedures for which animal samples were collected followed the recommendation of directive 2010/63/EU.

### 2.2 Animals and phenotypes

Phenotypic data from 481 ewes were collected between 2006 and 2012 in four flocks (*n* = 30, 268, 135 and 48, respectively) of Valle del Belice dairy sheep. The ewes were distributed in nine half‐sib families with an average size of 50 daughters per ram (ranging from 11 to 173 animals per half‐sib family). Some individuals across families were related through known pedigree relationships. The pedigree over three generations included 1,304 animals, of which 101 are rams and 823 dams.

Milk samples were collected at approximately monthly intervals, following an A4 recording scheme (ICAR, 2016). All ewes were milked manually twice a day. Milk yield (MY), computed as the sum of the morning and evening measurements, was recorded, and samples were collected to determine FY, PY, F% and P%. Milk composition (i.e., FY and PY) was determined by infrared spectrophotometry using Combifoss 6200 system (Foss Electric Hillerød). The F% and P% were calculated as weighted average of the morning and evening milking with respect to MY. The data available for the 481 ewes to be used for this study included a total of 5,586 test‐day (TD) records. A quality control (QC) of the phenotypic data was performed using S.A.S. version 9.4 (SAS Institute Inc., 2014): animals with lactation length >300 days, with less than three TD records within lactation, or with missing information on any milk traits (MY, FY, F%, PY and P%) were discarded. After QC, the data set consisted of 5,446 records for MY, 5,437 records for FY and F%, and 5,436 for PY and P% of 481 ewes (Table 1).

**TABLE 1:** Descriptive statistics for milk production traits

Traits	*N*	Mean ± *SD*	CV(%)	Min‐Max
MY (g)	5,446	1,367 ± 532	38.92	100–3,924
F% (%)	5,437	7.08 ± 1.06	14.97	2.53–15.78
FY (g)	5,437	94.80 ± 33.91	35.77	6–277
P% (%)	5,436	5.82 ± 0.65	11.17	2.32–11.60
PY (g)	5,436	78.93 ± 29.32	37.15	6–239

Abbreviations: CV, coefficient of variation; F%, Fat percentage; FY, Fat Yield; Min‐Max, minimum and maximum values; MY, Milk Yield; *N*, number of records; P%, Protein percentage; PY, Protein Yield; *SD*, standard deviation.

### 2.3 Genotypic data

Blood samples from 481 Valle del Belice ewes were collected in order to extract DNA. Genotyping was performed with Illumina OvineSNP50 (50k) BeadChip. Using GenomeStudio v2.0 software (Illumina), genotypes of 54,241 SNPs were available for each individual. The genomic positions of SNPs on chromosomes were updated to the ovine Oar_v4.0 genome assembly (https://www.ncbi.nlm.nih.gov/genome/?term=ovis+aries). QC was performed using PLINK v 1.07 (Purcell et al., 2007). Only SNPs located on autosomes were considered for further analyses. Animals and markers that fulfilled the following criteria were kept in the analysis: (a) call rate per individuals and per SNPs >95%, (b) minor allele frequency >2% and (c) no extreme deviation from Hardy‐Weinberg equilibrium (*p* <.001). After QC, 37,228 SNPs and 481 individuals were retained for further analyses.

### 2.4 Variance component estimation

The TD traits analysed as response variables were MY, F%, FY, P% and PY. Variance components and genetic parameters for each trait were estimated with GBLUP, assuming a repeatability TD animal model:

$$y=X\beta +Z_{1}u+Z_{1}\mathrm{pu}+Z_{2}\mathrm{pw}+Z_{3}h+e$$

where **y** was the TD record for the considered traits; **β** is a vector of the fixed effects, including parity (five levels), litter size (two levels, single or multiple born lambs), season of lambing (two levels, where the season of lambing was coded as 1 if ewe gave birth in the period January‐June, otherwise, it was coded as 2, as suggested by Riggio et al., 2007) and fortnights in milk FIM (22 levels); **u** is a vector (481) of the random animal's additive genetic effects $\left[{u∼(0,G\sigma }_{u}^{2})\right]$; **pu** is a vector (481) of the random permanent environmental effect of the individuals across lactations $\left[{pu∼(0,I\sigma }_{\mathrm{pu}}^{2})\right]$; **pw** is a vector (2,405) of the random permanent environmental effect of the individuals within parity class $\left[{pw∼(0,I\sigma }_{\mathrm{pw}}^{2})\right]$; **h** is a vector (180) of the random flock by test‐day (FTD) interaction (Ptak & Schaeffer, 1993) effects $\left[{h∼(0,I\sigma }_{h}^{2})\right]$; **e** is a vector of the random residual errors $\left[{e∼(0,I\sigma }_{e}^{2})\right]$; and **X**, **Z_1_**, **Z_2_** and **Z_3_** are the corresponding incidence matrices. The $\sigma_{u}^{2}$, $\sigma_{\mathrm{pu}}^{2}$, $\sigma_{\mathrm{pw}}^{2}$, $\sigma_{h}^{2}$ and $\sigma_{e}^{2}$ terms are the additive genetic, permanent environment (across and within lactation), FTD and residual error variances, respectively; and **G** and **I** are the additive genetic relationship and identity matrices, respectively. The **G** matrix is the GRM calculated using VanRaden's method 2 (VanRaden, 2008), with all SNPs across the genome. Our model included fortnights in milk (FIM) as fixed effect; but alternatively, we could have fitted days in milk (DIM) using spline curve. Preliminary analyses showed that the residuals for MY when fitting DIM as spline were basically the same as when using our model fitting FIM as fixed effect (Figure S1). The analysis was performed using ASREML v3.0 (Gilmour et al., 2009) using the generalized Moore‐Penrose inverse (Moore, 1920; Penrose, 1955) of the GRM.

### 2.5 Regional heritability mapping

In the RHM analysis, each chromosome (OAR) was divided into regions—or equivalently, windows—(throughout the paper both terms will be used and they will be interchangeable) of a predefined number of consecutive SNPs, and the variance attributable to each window estimated (i.e., the regional variance). In this study, a window size of 100 adjacent SNPs was used and the window was shifted every 50 SNPs so adjacent windows overlap by half. Because the number of SNPs varies across chromosomes, the last region at the end of each chromosome was excluded if it contained less than 25 SNPs.

Each genomic region or window was tested by fitting a linear mixed model accounting for all the fixed and random effects included when estimating the variance components, plus an additional random additive effect associated with the genomic region being tested. The regional genomic additive effect (**v**) assumed to be distributed as ${v∼(0,G_{v}\sigma }_{v}^{2})$, where $\sigma_{v}^{2}$is the additive genetic variance associated with the region and $G_{v}$is the regional GRM estimated in the same way as **G** but using only the SNPs located within the genomic region in question (Nagamine et al., 2012). The respective whole genomic and regional heritability were estimated as $h_{u}^{2}=\left(\sigma_{u}^{2}/\sigma_{p}^{2}\right)$ and $h_{v}^{2}=\left(\sigma_{v}^{2}/\sigma_{p}^{2}\right)$, where $\sigma_{p}^{2}$is the phenotypic variance equal to the sum of all variances (i.e., $\sigma_{p}^{2}=\sigma_{u}^{2}+\sigma_{v}^{2}+\sigma_{\mathrm{pu}}^{2}+\sigma_{\mathrm{pw}}^{2}+\sigma_{h}^{2}+\sigma_{e}^{2}$).

The statistical significance of the variance associated with a specific region was tested using a likelihood ratio test (LRT), which compares the log‐likelihood (logL) of the full model including $\sigma_{v}^{2}$ (H1) against that of the null model without $\sigma_{v}^{2}$ (H0). The LRT for a specific region is equal to 2*(logL(H1)‐logL(H0)), and it was assumed to follow a mixture of $\frac{1}{2}\chi_{\left(1\right)}^{2}$ and $\frac{1}{2}\chi_{\left(0\right)}^{2}$ distributions (Self & Liang, 1987). Since a total of 827 windows (with consecutive ones overlapping by half) were tested, the Bonferroni correction to account for multiple testing was done assuming 827/2 independent tests. Hence, after Bonferroni correction, the LRT thresholds for genome‐wide 5%‐significance and suggestive significance (i.e., defined as the level at which one false positive per genome scan is expected) were 13.48 and 9.20, corresponding to *p*‐values of *p* < 1.21 × 10^−4^ (−log_10_(*p*) of 3.92) and *p* < 2.41 × 10^−3^ (−log_10_(*p*) of 2.62), respectively. The RHM analysis was performed using an in‐house REML software, which does not require the inverse of the GRMs as suggested by Lee and van der Werf (2006) and implemented in popular software such as GCTA (Yang et al., 2011) and MTG2 (Lee & van der Werf, 2016).

### 2.6 Gene annotation

To investigate if the significant SNPs detected in this study mapped within genes or previously reported QTL for relevant traits, we searched them in NCBI Genome Data Viewer for Ovis aries v4.0 genome assembly (https://www.ncbi.nlm.nih.gov/genome/gdv/browser/?context=genome&acc=GCF_000298735.2) and in the SheepQTLdb (https://www.animalgenome.org/cgi‐bin/QTLdb/OA/index) for milk production QTL, respectively. To investigate the biological function and the phenotypes that are known to be affected by each annotated gene, we also conducted a comprehensive literature search, including information from other species.

## 3 RESULTS AND DISCUSSION

### 3.1 Variance component estimates

Summary statistics for milk production traits are reported in Table 1. The coefficients of variation (CV) for milk, fat and protein yields were between 36% and 39%, with those for fat and protein percentages being considerably lower (i.e., 15% and 11.2%, respectively). This is in line with the coefficients of variation found in other studies (Hamann et al., 2004; Oravcová et al., 2005).

Variance components, heritability and within and across lactation repeatability estimates as well as proportions of variance due to FTD are shown in Table 2. Heritability estimates for milk yield and milk composition traits were low and varied between 0.06 and 0.15. Our heritabilities were in the range (i.e., 0.04–0.34) reported for other sheep breeds using TD models (Marina et al., 2020) but lower than those (i.e., 0.31–0.61) using lactation models (Usai et al., 2019), when using the GRM. A similar trend was observed when comparing our estimates to those obtained using pedigree, with our heritability estimates for test‐day milk yield, as well as fat and protein percentage being in the range reported in literature, which is between 0.10 and 0.24 for milk yield and between 0.06 and 0.39 for fat and protein percentage, respectively (Barillet et al., 2001; El‐Saied et al., 1998; Oravcová et al., 2005; Othmane et al., 2002). However, Hamann et al. (2004) reported heritability estimates of 0.15 for fat and protein yield, which are higher than the estimates found in the present study. Still, our estimates were lower than those reported in literature when using lactation models and pedigree information (for a review, Carta et al., 2009). It is important to highlight that our heritability estimates may be partially biased by an over‐parametrization of our analysis to take into account the repeated measurements both within and across lactations. Serrano et al. (2003) in a comparison between mean lactation and TD approaches have reported that using repeatability TD models, the heritability estimates tend to be lower, as there is an increase of the permanent environmental variance and a decrease of the genetic component, whereas the residual variance does not change. Our repeatability estimates were generally higher than heritabilities and significant, ranging between 0.14 and 0.24 across lactations, with the highest estimates being for fat and protein percentage, and between 0.23 and 0.39 within lactation, with the highest estimates being for milk, fat and protein yields. These estimates were higher than those reported by Komprej et al. (2009) in Slovenian sheep, ranging between 0.03 and 0.11 across lactations and between 0.01 and 0.13 within lactation.

**TABLE 2:** Variance components, heritabilities and repeatabilities for milk production traits

Traits	$\sigma_{u}^{2}$	$\sigma_{\mathrm{pu}}^{2}$	$\sigma_{\mathrm{pw}}^{2}$	$\sigma_{\mathrm{ftd}}^{2}$	$\sigma_{P}^{2}$	*h*^2^ ± *SE*	r_acr_ ± *SE*	r_wit_ ± *SE*	FTD^2 ^± *SE*
MY	24,811	16,967	52,716	96,410	254,968	0.10 ± 0.03	0.16 ± 0.02	0.37 ± 0.03	0.38 ± 0.03
F%	0.1276	0.0734	0.0620	0.4530	1.1201	0.11 ± 0.03	0.18 ± 0.03	0.23 ± 0.03	0.40 ± 0.03
FY	103	66	244	369	1,070	0.06 ± 0.03	0.16 ± 0.02	0.39 ± 0.03	0.34 ± 0.03
P%	0.0588	0.0306	0.0257	0.1269	0.3786	0.15 ± 0.03	0.24 ± 0.02	0.30 ± 0.03	0.33 ± 0.03
PY	71	44	168	333	805	0.09 ± 0.03	0.14 ± 0.02	0.35 ± 0.03	0.41 ± 0.03

Abbreviation: $\sigma_{\mathrm{ftd}}^{2}$, variance of flock‐test‐day interaction; $\sigma_{P}^{2}$, phenotypic variance; $\sigma_{\mathrm{pu}}^{2},$ permanent environmental variance across lactations; $\sigma_{\mathrm{pw}}^{2}$, permanent environmental variance within lactation; $\sigma_{u}^{2}$, additive genetic variance; F%, Fat percentage; FTD^2^, ratio of the flock‐test‐day interaction and the phenotypic variance; FY, Fat Yield; *h* ^2^, heritability; MY, Milk Yield; P%, Protein percentage; PY, Protein Yield; r_acr_, repeatability across lactations; r_wit_, repeatability within lactation; *SE*, standard error.

The proportion of variation explained by FTD was large for all milk production traits, ranging between 0.33 and 0.41. Flock by test‐day interaction represents the different technologies of sheep breeding and feeding manners among flocks including temporary events within the flock on the day of recording or some days before (Komprej et al., 2009). Management of the Valle del Belice breed is indeed characterized by the enormous variability, part of which is due to the fact that most of the farmers milk ewes by hand, but some of the farms use a milking machine. Furthermore, the lambing system is different from the one adopted in other Mediterranean regions (Carta et al., 2009), with Valle del Belice ewes lambing all year long, starting in July and finishing in the following June, with few lambings in May and June (Finocchiaro et al., 2005). Moreover, sheep are fed natural pastures and fodder crops; supplementation, consisting of hay and sometimes concentrates, is occasionally supplied, for example at the end of gestation (Cappio‐Borlino et al., 1997). The grazing possibilities and the chemical and nutritional composition of the feed change annually and also differ among areas. This probably explains why FTD explained the largest part of phenotypic variance among all random effects. A similar result was reported by Bittante et al. (2017), showing a very high effect of individual flock/date representing almost 75% of the total phenotypic variance of milk yield, and 30%–35% for milk quality traits.

### 3.2 Regional heritability mapping analysis

**FIGURE 1:**
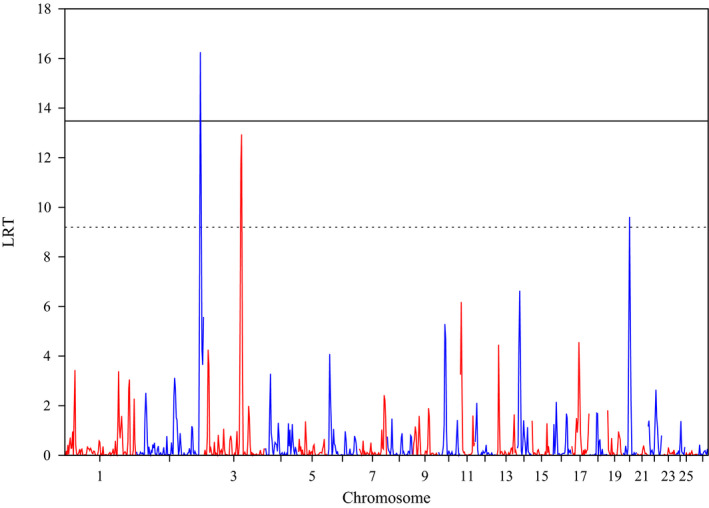
Plot of the likelihood ratio test (LRT) across the genome for fat percentage (F%). The 5% genome‐wide Bonferroni corrected threshold (LRT > 13.48; solid line) and the suggestive threshold (i.e., one expected false positive per genome scan; LRT > 9.20; dashed line) are also shown

**FIGURE 2:**
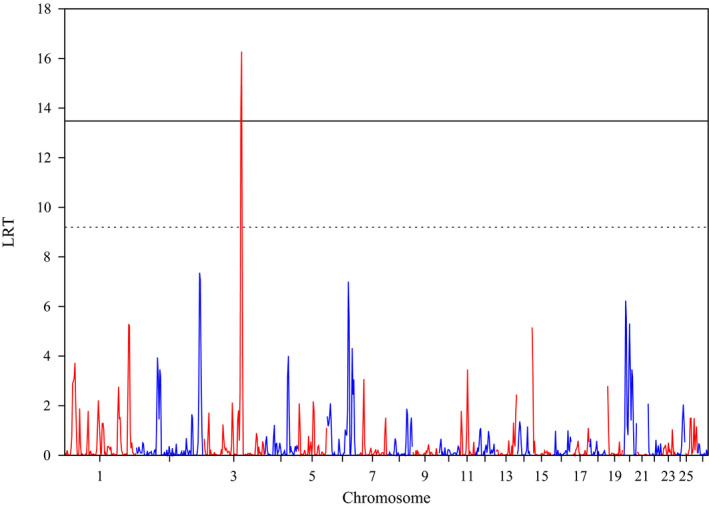
Plot of the likelihood ratio test (LRT) across the genome for protein percentage (P%). The 5% genome‐wide Bonferroni corrected threshold (LRT > 13.48; solid line) and the suggestive threshold (i.e., one expected false positive per genome scan; LRT > 9.20; dashed line) are also shown

The results of the RHM analysis using 100 SNP window size for milk production traits are shown in Table 3 and Figures 1 and 2. A region on OAR2 was found significant at the genome‐wide level (LRT = 16.25) for F%, with an overlapping window reaching the suggestive significance threshold (LRT = 10.98). Three more regions (two consecutive ones on OAR3 and one on OAR20) reached the suggestive significant threshold for the same trait. However, the same two consecutive regions on OAR3 reached the genome‐wide significance threshold for P%. The regions on OAR2 and OAR3 were previously identified by standard GWAS for milk production traits in other dairy sheep breeds, whereas the region on OAR20 only partially overlapped a known QTL for fat percentage. No other regions were found significant at either the genome‐wide or the suggestive thresholds for the other traits. The reduced number of significant regions identified might be due to the small sample size, which can be a possible cause of power reduction to detect significant regions.

**TABLE 3:** Regional heritability (*h* ^2^v) for milk production traits for regions significant at either the 5% genome‐wide Bonferroni corrected level (LRT > 13.48) or the suggestive level (i.e., one expected false positive per genome scan; LRT > 9.20)

Trait	OAR	Region	SNP and position (in bp)		LRT	*h*^2^v
			Start	End		
*F* %	2	83	rs413324492 234,715,088	rs401097503 240,388,878	16.25^g^	0.03
	2	84	rs407871693 237,161,006	rs412038888 243,300,137	10.98	0.02
	3	48	rs412220800 134,166,768	rs419412283 139,724,363	11.71	0.02
	3	49	rs407496519 137,065,870	rs418178732 142,443,170	12.94	0.02
	20	9	rs429947991 23,043,970	rs399189470 30,066,632	9.61	0.02
P%	3	48	rs412220800 134,166,768	rs419412283 139,724,363	13.86^g^	0.03
	3	49	rs407496519 137,065,870	rs418178732 142,443,170	16.27^g^	0.03

Note

g: region significant at genome‐wide level.

The genomic regions detected on OAR3 reported several QTLs identified in different studies for the considered traits. In particular, a QTL (ID = 13905) for P% was found by Gutiérrez‐Gil et al. (2009) on OAR3 in a commercial population of Spanish Churra sheep. The same authors reported QTLs (ID = 13915 and 13917) on OAR2 and OAR20, respectively, which showed suggestive significant associations with F% (Gutiérrez‐Gil et al., 2009). Several GWAS studies for milk production traits on Spanish Churra sheep reported several QTLs (IDs = 57707, 57708, 57739, 57741, 17200) on OAR3 which overlapped with our regions significantly associated with F% and P% (García‐Gámez, Gutiérrez‐Gil, Sahana, et al., 2012; García‐Gámez et al., 2012, 2013).

Several genes, pseudogenes and tRNA genes have been found in the identified regions (Table S1), with the most interesting being on OAR3. Among these, *LALBA* (*α‐lactalbumin*) on OAR3 is considered a strong functional candidate gene affecting the traits analysed. Alpha‐lactalbumin is a major whey protein that forms a subunit of the lactose synthase binary complex. Because lactose synthase is necessary for the production of lactose and the subsequent movement of water into the mammary secretory vesicles, this enzyme is critical in the lactation control and secretion of milk (García‐Gámez, Gutiérrez‐Gil, Sahana, et al., 2012). Previous studies on LALBA‐deficient mice have shown the influence of this enzyme on the protein and fat concentration in milk, with homozygous mutant mice producing highly viscous milk that is very rich in fat and protein and devoid of alpha‐lactalbumin and lactose (Stinnakre et al., 1994). Polymorphisms in the *LALBA* gene were studied in the 1990s as possible markers related to milk production in dairy species. *LALBA* gene was identified as candidate gene that influenced milk protein percentage in Spanish Churra sheep (Gutiérrez‐Gil et al., 2009). García‐Gámez, Gutiérrez‐Gil, Sahana, et al. (2012) hypothesized that the Ala27Val substitution at α‐lactalbumin would reduce the synthesis of lactose and water secretion into the milk causing an increase in milk fat and protein concentration in the same breed. Moreover, similar effects were also obtained by Padilla et al. (2018) in Merinos sheep. This study identified marker rs399070200, located in the third intron of this gene, as the SNP with the most highly significant association, detected on OAR3 both for P% and F%. Moreover, on OAR3, we found Aquaporins (AQPs) genes, in particular, *AQP6*, *AQP5* and *AQP2* (in bold in Table S1). This is a family of ubiquitous membrane proteins involved in the transport of water and wide range of solutes (Gomes et al., 2009). A functional role for some members of this family during the production and secretion of bovine milk was confirmed in an immuno‐histochemical study conducted by Mobasheri et al. (2008). Finally, we observed genomic regions containing olfactory receptor (OR) family genes (19 on OAR3 and 62 on OAR20 indicated as “LOC” in bold in Table S1). Olfactory receptors detect and identify a wide range of odours and chemosensory stimuli, are necessary to find food, detect mates and offspring, to recognize territories and avoid dangers.

In this study, we used RHM approach instead of association analysis from a standard GWAS to detect genomic regions underlying milk production traits in Valle del Belice dairy sheep. Literature suggests that our method of choice performs better than a standard GWAS, especially when associated SNPs do not have large enough effect to be declared significant at the genome‐wide level. Moreover, the RHM method may be able to reduce the false discovery rate, a common problem with GWAS method in populations that are characterized by a high inbreeding coefficient (Manenti et al., 2009) using nearby SNP information and the summation of SNP effects on a window. In principle, estimating the trait heritability for chosen regions allows integration of the variance contributed by both rare and common variants into a single estimate of additive variance. Because the RHM combines different sources of variation within the region, it potentially allows the identification of loci that cannot be found by standard GWAS. Therefore, RHM should have greater power than a standard GWAS analysis to map genomic regions that contribute variance due to the segregation of several common or rare variants while retaining similar power to map loci segregating for a single common variant.

## 4 CONCLUSION

We have successfully detected genomic regions associated with fat and protein percentage in the Valle del Belice sheep breed, using RHM approach. Our results confirmed the role of *LALBA* and *AQP* genes, on OAR3, as candidate genes for milk production traits in sheep. Moreover, heritability estimates for milk production traits ranged between 0.06 and 0.15, with repeatabilities being between 0.14 and 0.24 across lactations and between 0.23 and 0.39 within lactation. A substantial effect of flock‐test‐day on milk production traits was also estimated. Further studies to refine heritability estimates might be needed in the future using bigger sample size.

## CONFLICT OF INTEREST

The authors declare that there is no conflict of interest regarding the publication of this article.

## Supporting information

Fig S1

Table S1

## Data Availability

The data that support the findings of this study are available on request from the corresponding author.
