# Supplementary figures and images for: Detection of genomic regions underlying milk production traits in Valle del Belice dairy sheep using regional heritability mapping
Source: J Anim Breed Genet. 2021 May 20;138(5):552–61. doi: 10.1111/jbg.12552 (PMC8453569; doi:10.1111/jbg.12552)

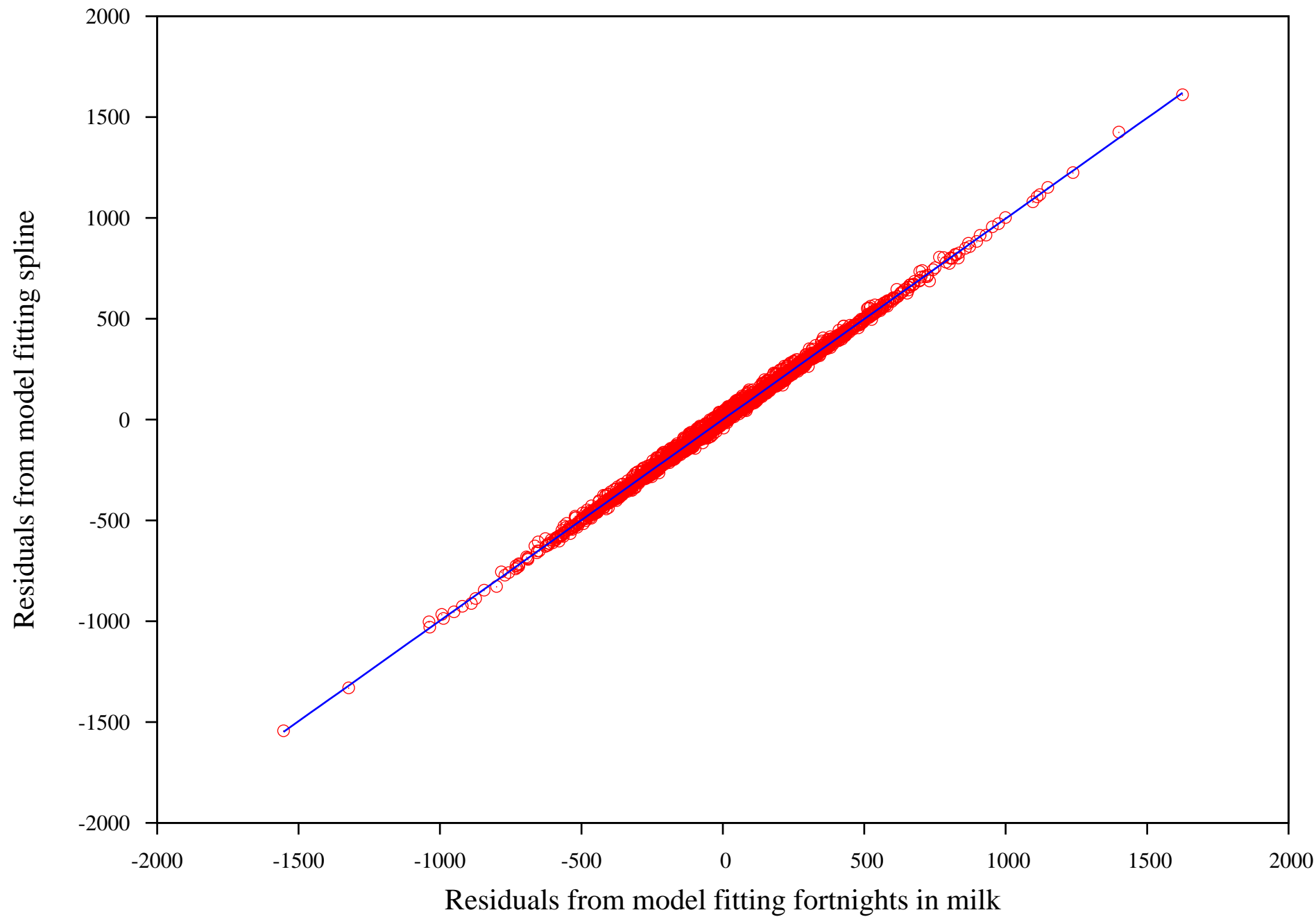

Supplement: Supplementary file 1 — Fig S1 [file JBG-138-552-s002.pdf]
