# Supplementary material for: Detection of genomic regions underlying milk production traits in Valle del Belice dairy sheep using regional heritability mapping
Source: J Anim Breed Genet. 2021 May 20;138(5):552–61. doi: 10.1111/jbg.12552 (PMC8453569; doi:10.1111/jbg.12552)
Supplement: Supplementary file 2 — Table S1 [file JBG-138-552-s001.docx]

| CHR | Start | End | N° SNP | Gene |
| --- | --- | --- | --- | --- |
| 2 | 234715088 | 240388878 | 116 | SPOCD1, ADGRB2, COL16A1, PEF1, HCRTR1, TINAGL1, LOC101107259, SERINC2, LOC105608219, FABP3, ZCCHC17, TRNAQ-CUG, SNRNP40, NKAIN1, PUM1, SDC3, LOC101108033, LAPTM5, MATN1, LOC105608216, LOC105611135, LOC105608214, PTPRU, MECR, SRSF4, LOC105611150, TMEM200B, EPB41, OPRD1, TRNAE-UUC, YTHDF2, GMEB1, LOC101123533, TAF12, RAB42, LOC101102543, TRNAU1AP, RCC1, LOC105608205, PHACTR4, LOC101110400, MED18, SESN2, TRNAG-CCC, LOC101110916, PTAFR, LOC101111170, LOC101104811, LOC101111434, EYA3, XKR8, RPA2, THEMIS2, LOC101112204, PPP1R8, STX12, FAM76A, LOC101106571, IFI6, FGR, AHDC1, WASF2, LOC105606955, GPR3, CD164L2, FCN3, MAP3K6, SYTL1, TMEM222, LOC105606957, LOC101108028, WDTC1, SLC9A1, LOC105606958, FAM46B, LOC105606960, TRNP1, KDF1, NUDC, NR0B2, GPN2, SFN, ZDHHC18, PIGV, LOC105611280, ARID1A,  LOC106990911, RPS6KA1, LOC101110394, HMGN2, DHDDS, LIN28A, LOC105606964, ZNF683, AIM1L, UBXN11, SH3BGRL3, CEP85, CATSPER4, LOC105606966, CNKSR1, ZNF593, FAM110D, PDIK1L, TRIM63, LOC101117060, SLC30A2, EXTL1, PAFAH2, STMN1, LOC106990973, PAQR7, AUNIP, MTFR1L, SEPN1, MAN1C1, LDLRAP1 |
| 2 | 237161006 | 243300137 | 119 | PTPRU, MECR, SRSF4, LOC105611150, TMEM200B, EPB41, OPRD1, TRNAE-UUC, YTHDF2, GMEB1, LOC101123533, TAF12, RAB42, LOC101102543, TRNAU1AP, RCC1, LOC105608205, PHACTR4, LOC101110400, MED18, SESN2, TRNAG-CCC, LOC101110916, PTAFR, LOC101111170, LOC101104811, LOC101111434, EYA3, XKR8, RPA2, THEMIS2, LOC101112204, PPP1R8, STX12, FAM76A, LOC101106571, IFI6, FGR, AHDC1, WASF2, LOC105606955, GPR3, CD164L2, FCN3, MAP3K6, SYTL1, TMEM222, LOC105606957, LOC101108028, WDTC1, SLC9A1, LOC105606958, FAM46B, LOC105606960, TRNP1, KDF1, NUDC, NR0B2, GPN2, SFN, ZDHHC18, PIGV, LOC105611280, ARID1A, LOC106990911, RPS6KA1, LOC101110394, HMGN2, DHDDS, LIN28A, LOC105606964, ZNF683, AIM1L, UBXN11, SH3BGRL3, CEP85, CATSPER4, LOC105606966, CNKSR1, ZNF593, FAM110D, PDIK1L, TRIM63, LOC101117060, SLC30A2, EXTL1, PAFAH2, STMN1, LOC106990973, PAQR7, AUNIP, MTFR1L, SEPN1, MAN1C1, LDLRAP1, TMEM57, LOC101114847, TMEM50A, RSRP1, SYF2, RUNX3, CLIC4, LOC106990974, LOC101116025, LOC105606970, SRRM1, TRNAE-UUC, TRNAS-GGA, RCAN3, NIPAL3, STPG1, GRHL3, TRNAC-GCA, IFNLR1, IL22RA1, MYOM3, SRSF10, PNRC2, LOC101118928, CNR2, FUCA1, HMGCL, GALE, LYPLA2, PITHD1, TCEB3, LOC105606974, RPL11, ID3, E2F2, ASAP3, TCEA3, LOC106990852, LOC105606976, ZNF436, HNRNPR, TRNAV-CAC, HTR1D, LUZP1, KDM1A, C2H1orf234,  LACTBL1, EPHB2, C1QB, LOC101123446, C1QA, EPHA8, ZBTB40 |
| 3 | 134166768 | 139724363 | 110 | SCN8A, TRNAE-UUC, SLC4A8, GALNT6", TRNAG-CCC, CELA1, BIN2, SMAGP, DAZAP2, LOC106990996", TRNAR-CCU, POU6F1, LOC101115112, TFCP2, CSRNP2, LETMD1, LOC105610932, LOC101115614, LOC105610931, LOC101116975, LOC101117657, TMPRSS12, ATF1, TRNAE-UUC, DIP2B, LOC101118348, TRNAC-ACA, LOC105610928, LARP4, FAM186A, LIMA1, CERS5, LOC101119459, GPD1, SMARCD1, ASIC1, LOC105612625, ***AQP6, AQP5, AQP2***, FAIM2, BCDIN3D, LOC105612627, NCKAP5L, TMBIM6, LOC105612628, FMNL3, PRPF40B, FAM186B, MCRS1, KCNH3, SPATS2, DNAJC22, C1QL4, ROAP, LOC101119463, PRPH, LOC101119713, LOC105614728, LOC101119975, LOC106990101, LMBR1L, TRNAC-GCA, DHH, RHEBL1, KMT2D, PRKAG1, DDN, WNT1, WNT10B, ARF3, TRNAN-GUU, FKBP11, CCDC65, RND1, DDX23, CACNB3, ADCY6, CCNT1, KANSL2, ***LALBA***, LOC101121506, LOC101122268, LOC101122513, LOC101122776, LOC101123028, LOC101123287, LOC101123547, LOC101101975, LOC101102226, LOC101102472, LOC101102718, LOC101102972, LOC101103226, LOC101103465, LOC101103713, C3H12orf54, LOC101103970, LOC101104481, LOC101104739, LOC101104986, LOC101105490, LOC101105739, ZNF641, LOC101105997, LOC101106252, LOC105611712, LOC101106515, LOC101106764, CCDC184, ASB8, LOC105611714, PFKM, SENP1, COL2A1, TMEM106C, VDR, LOC105611708, HDAC7, SLC48A1, RAPGEF3, ENDOU, RPAP3, LOC105611709, PCED1B, AMIGO2, TRNAG-CCC,  LOC106990997, SLC38A4, LOC101108564 |
| 3 | 137065870 | 142443170 | 115 | CCNT1, KANSL2, LALBA**, LOC101121506, LOC101122268, LOC101122513, LOC101122776, LOC101123028**, LOC101123287**, LOC101123547, LOC101101975, LOC101102226, LOC101102472, LOC101102718, LOC101102972, LOC101103226, LOC101103465**, LOC101103713, C3H12orf54, **LOC101103970, LOC101104481, LOC101104739**, LOC101104986, LOC101105490**, LOC101105739**, ZNF641, LOC101105997, LOC101106252, LOC105611712, LOC101106515, LOC101106764, CCDC184, ASB8, LOC105611714, PFKM, SENP1, COL2A1, TMEM106C, VDR, LOC105611708, HDAC7, SLC48A1, RAPGEF3, ENDOU, RPAP3, LOC105611709, PCED1B, AMIGO2, TRNAG-CCC, LOC106990997, SLC38A4, LOC101108564, SLC38A2, SLC38A1, SCAF11, ARID2, ANO6, TRNAS-GGA,  LOC105611661, NELL2, LOC105613432 |
| 20 | 23043970 | 30066632 | 115 | TFAP2B, LOC106991807, PKHD1, LOC105603743, IL17A, IL17F, MCM3, LOC101104694, TRNAE-UUC, LOC105603744, PAQR8, EFHC1, LOC101118825, TRAM2, TMEM14A, GSTA1-1, TRNAG- CCC, LOC101106291, LOC101106720, LOC101106976, TRNAR-UCU, LOC101107232, ICK, FBXO9, GCM1, ELOVL5, LOC101108696, OVAR-DRB, LOC105612264, LOC101119856, LOC105603927, LOC101109220, DQB, LOC101120118, LOC101109492, OVAR-DRB3, DQA, LOC101120871, LOC106990179, LOC101109747, BTNL2, LOC101110006, LOC101110277, LOC101121379, LOC105603751, LOC101121635, LOC101110546, LOC105603754, LOC105603755, C20H6orf10, LOC106991808, LOC105603929, LOC101111058, LOC101122142, LOC106991799, NOTCH4, "GPSM3, PBX2, AGER, RNF5, AGPAT1, EGFL8, PPT2, PRRT1, FKBPL, ATF6B, LOC105603761, TNXB, LOC101123159, LOC105612706, LOC101123419, LOC105612707, LOC105603760, LOC101123672, STK19, DXO, SKIV2L, NELFE, CFB, C2, ZBTB12, EHMT2, SLC44A4, NEU1, LOC101116687, LOC105603930, HSPA1A, LOC494436, HSPA1L, LSM2, VARS, LOC106991809, VWA7, SAPCD1, MSH5, CLIC1, DDAH2, C20H6orf25, LY6G6C, LY6G6D, LOC101119591, LY6G6F, ABHD16A, LOC105603765, LY6G5C, LY6G5B, CSNK2B, GPANK1, C20H6orf47, APOM, BAG6, PRRC2A, NCR3, AIF1, LST1, LTB, TNF, LTA, LOC105603766, NFKBIL1, ATP6V1G2, DDX39B, MCCD1, LOC106990117, LOC105603776, LOC101105367, LOC101105609, LOC106991789, LOC105614324, LOC105603775, MICA, POU5F1, TCF19, CCHCR1, PSORS1C2, CDSN, C20H6orf15, LOC101106374, LOC105603772, LOC101109651, MUC21, DPCR1, LOC106990499, SFTA2, VARS2, GTF2H4, DDR1, LOC105603769, IER3, FLOT1, TUBB, MDC1, NRM, PPP1R18, DHX16, C20H6orf136, ATAT1, MRPS18B, PPP1R10, RPP21, GNL1, PRR3, ABCF1, LOC101106373, LOC101107908, LOC101108171, LOC101108432, LOC101110710, LOC106991793, LOC105603777, OLA-I, LOC105603778, LOC101110973, LOC101111233, TRIM26, TRIM15, TRIM10, TRIM40, TRIM31, TRNAL- CAA, RNF39, PPP1R11, LOC105603779, ZNRD1, LOC105603780, ZFP57, MOG, GABBR1, LOC101113705**, LOC101111325**, LOC101113965, **LOC101111587, LOC101114220, LOC101111839, LOC101112095, LOC101114473, LOC101112357, LOC101114731**, LOC101114983, LOC101112607**, LOC101112857**, LOC101115237, **LOC101113109, LOC101113370, LOC101113633**, LOC101113894, **LOC105613647, LOC106991794, LOC101115488, LOC101114399, LOC101114653, LOC101114912, LOC101115167, LOC101115411, LOC101115662**, LOC101115916, LOC101116185, **LOC101116436, LOC101116688, LOC101116944**, LOC101115997, LOC101117202, **LOC101117457, LOC101117709, LOC101117974, LOC101118231**, LOC101118485, **LOC101118741, LOC101116262**, LOC101119001, **LOC101119263, TRNAG-ACC, LOC101116514, LOC101119515, LOC101116772, LOC101119769, LOC101117201, LOC101120028, LOC101120282, LOC101120531**, LOC101120786**, LOC101117455, LOC101121048, LOC101121301, LOC101121555, LOC101117708**, LOC101122062, LOC101122314, LOC101122560, LOC101122821, ZNF311, TRNAL-AAG, TRNAE-CUC, TRNAF-GAA, LOC105603785, TRNAM-CAU, TRNAK-UUU, TRNAM-CAU", TRNAL-AAG, TRNAL-CAA, LOC105603786, TRNAL-CAA, TRNAR-CCG, TRNAA-AGC, TRNAA-CGC, TRNAF-GAA, TRNAA-UGC, TRNAK-UUU, TRNAR-CCG, TRNAA-AGC, TRNAM-CAU, TRNAS-AGA, TRNAS-AGA, TRNAQ-CUG, ZBED9, TRNAK- UUU, TRNAT-UGU, TRNAR-UCG, TRNAI-UAU, GPX5, GPX6, TRNAA-AGC, TRNAM-CAU, TRNAT-UGU, TRNAT-UGU, LOC101123333, TRNAF-GAA, ZSCAN23, ZSCAN31, PGBD1, LOC105603790, NKAPL, ZSCAN9, TRNAS-GCU, LOC106991791, LOC101104612, ZKSCAN8, LOC105603808, ZSCAN16, ZNF165, LOC105603933, LOC105603806, LOC105603804, LOC106991795, LOC105603935, LOC105603803, LOC105603936, LOC105603937, LOC105603802, LOC105603801, LOC105603938, LOC105603800, LOC105603799, LOC105603939, LOC105603798, LOC101103354, LOC101102593, LOC105603940, LOC105612194, LOC105603796, TRNAG-GCC, TRNAM-CAU, LOC101119941, LOC106990414, LOC101120961, LOC101123158, LOC101102344, LOC101119679, LOC101118999, LOC101123417, LOC101123670, LOC101122637, LOC101120447, LOC101118484, LOC101120702, LOC101119426, TRNAM-CAU, TRNAQ-UUG, TRNAQ-CUG, TRNAM-CAU, TRNAV-AAC, TRNAT-AGU, TRNAA-AGC, TRNAL-UAA, LOC105603793, TRNAI-AAU, TRNAI-AAU, TRNAT-AGU, TRNAV-AAC, TRNAS-CGA, TRNAR-ACG, TRNAK-UUU, TRNAI-AAU, TRNAF-GAA, TRNAI-UAU, TRNAT-CGU, TRNAL-CAA, TRNAL-CAA, TRNAM-CAU, TRNAK-UUU, TRNAD-GUC, TRNAK-UUU, TRNAL-CAA, TRNAR-UCU, TRNAS-AGA, TRNAQ-CUG, TRNAS-UGA, TRNAS-AGA, TRNAM-CAU, TRNAS-AGA, TRNAS-AGA, TRNAQ-CUG, TRNAS-UGA, TRNAD-GUC, TRNAS-AGA, TRNAS-AGA, TRNAS-AGA, ZNF184, ZNF391 |

**Supplementary Table S1. Genes within the significant regions identified by regional heritability mapping for fat and protein percentage**
